# Supplementary material for: Complexome profiling of the Chlamydomonas psb28 mutant reveals TEF5 as an early PSII assembly factor
Source: Plant Cell. 2025 Mar 17;37(6):koaf055. doi: 10.1093/plcell/koaf055 (PMC12164590; doi:10.1093/plcell/koaf055)
Supplement: koaf055_Supplementary_Data [file koaf055_supplementary_data.zip › psb28_interactive.zip!/plots/intensity/qualityPlots/peptides/_Cre01.g054500.t1.2.html]

### Cre01.g054500.t1.2

Trivial name:   
SeqCoverage [median]: 46.50  
Mass: 112.35 kDa  
  
 

| Sequence | MissedCleavage | Mass [Da] | wtIntensity | muIntensity | Score |
| --- | --- | --- | --- | --- | --- |
| APADAAAVAGTHQEIDVGGVAEALTTASK | 0 | 2720.36 | 37644.67 | 104414.84 | 213.05 |
| AQMFDSLSSMANIAGYR | 0 | 1860.85 | 32433.31 | 93477.36 | 159.18 |
| AVTEATQHFER | 0 | 1287.62 | 126120.62 | 275130.27 | 198.62 |
| DHKKDTGPVDLYEPTKR | 3 | 1998.02 | 0.00 | 3353.43 | 95.20 |
| DTGPVDLYEPTK | 0 | 1333.64 | 422.29 | 572.64 | 103.31 |
| DTGPVDLYEPTKR | 1 | 1489.74 | 107765.35 | 152881.12 | 170.95 |
| EGAVLVSYLYPAR | 0 | 1436.77 | 30992.11 | 2429.19 | 210.08 |
| ELVEALAK | 0 | 871.50 | 13097.34 | 12839.46 | 128.57 |
| EMSPAFIEAEMK | 0 | 1381.63 | 30832.62 | 41615.92 | 220.82 |
| EMSPAFIEAEMKLFAAQAK | 1 | 2111.04 | 0.00 | 1399.91 | 49.54 |
| ENAPGER | 0 | 771.35 | 18760.39 | 33541.40 | 138.83 |
| FGIHPVAGR | 0 | 952.52 | 1245.30 | 13377.93 | 104.76 |
| FLNGQITAAGR | 0 | 1146.61 | 2182.48 | 24035.92 | 191.40 |
| GAGEYSQFTDAEYASAGATLVDSTEAMGADIVLR | 0 | 3465.59 | 770.26 | 0.00 | 46.31 |
| GAPLLITK | 0 | 811.52 | 21143.07 | 52744.45 | 149.40 |
| GHFRIDHADPVIR | 1 | 1531.80 | 0.00 | 6263.68 | 94.73 |
| GSLVLEDGELR | 0 | 1186.62 | 16095.65 | 21493.17 | 127.40 |
| HLTDKGISVK | 1 | 1096.62 | 24435.24 | 86092.97 | 148.28 |
| IDHADPVIR | 0 | 1034.55 | 14081.22 | 20123.59 | 160.11 |
| IKEGAVLVSYLYPAR | 1 | 1677.95 | 22148.94 | 33954.65 | 100.68 |
| KDTGPVDLYEPTKR | 2 | 1617.84 | 0.00 | 6379.74 | 65.04 |
| KQTDALLAR | 1 | 1014.58 | 14943.41 | 22908.05 | 135.71 |
| LFAAQAK | 0 | 747.43 | 23789.54 | 52996.43 | 105.42 |
| LLLSAGPFTGGPK | 0 | 1256.71 | 112752.35 | 172204.23 | 179.42 |
| MAGQSSTLYANNISK | 0 | 1583.76 | 42289.41 | 66710.34 | 263.89 |
| MLDMFK | 0 | 783.37 | 39643.20 | 57913.73 | 97.60 |
| NKELVEALAK | 1 | 1113.64 | 47059.94 | 110730.71 | 213.80 |
| QTDALLAR | 0 | 886.49 | 21723.50 | 28475.78 | 133.60 |
| RVALSPAAVAGLLK | 1 | 1364.85 | 18643.94 | 33196.41 | 109.84 |
| SDPLSLPGK | 0 | 912.49 | 58645.94 | 69133.86 | 127.46 |
| SKQVFFMK | 1 | 1013.54 | 697.14 | 888.27 | 89.70 |
| SKQVFFMKR | 2 | 1169.64 | 1236.24 | 8211.43 | 106.20 |
| SLGAIVR | 0 | 714.44 | 55475.91 | 101765.84 | 136.89 |
| SLPNVILGGYGMTAK | 0 | 1519.81 | 64896.27 | 178079.60 | 153.52 |
| SMGAEFLTVDIQEEGESGTGYSK | 0 | 2434.08 | 0.00 | 4666.79 | 107.06 |
| TMGAGYAGAENPVFYKPNTFMFLGDAK | 1 | 2896.36 | 7212.05 | 2468.22 | 82.64 |
| TPNRVTVVGYTDMPSR | 1 | 1791.89 | 37173.70 | 90472.77 | 153.72 |
| VALSPAAVAGLLK | 0 | 1208.75 | 42968.13 | 148515.50 | 197.21 |
| VFDTRTAVKEQAK | 2 | 1491.80 | 0.00 | 968.11 | 72.09 |
| VIAYLGDFIGAVTMTGSAIAFGK | 0 | 2301.21 | 939.22 | 0.00 | 88.94 |
| VIIVPGYGMAVANAQYPVAELAK | 0 | 2373.28 | 40030.59 | 0.00 | 114.88 |
| VLVIGGGVAGLAAVGAAK | 0 | 1521.92 | 33190.17 | 20235.22 | 139.86 |
| VNETLGIK | 0 | 872.50 | 8907.11 | 15436.85 | 118.06 |
| VNETLGIKA | 1 | 943.53 | 56073.39 | 145702.42 | 219.19 |
| VRPPALEEVNK | 1 | 1250.70 | 72807.60 | 105255.07 | 156.01 |
| VRPPALEEVNKIK | 2 | 1491.88 | 3734.16 | 33177.87 | 171.57 |
| VTVVGYTDMPSR | 0 | 1323.65 | 83065.68 | 92684.35 | 213.25 |
| WPPPPGVIPQANTK | 0 | 1500.81 | 3346.16 | 1598.60 | 105.86 |
|  |  |  | 1391415.6 | 2550518.1 |  |
